# Nicotine dependence and quitting stages of smokers in Nepal: A community based cross-sectional study

**DOI:** 10.1371/journal.pone.0266661

**Published:** 2022-04-08

**Authors:** Janaki Kumari Timilsina, Bimala Bhatta, Amrit Devkota

**Affiliations:** 1 Bachelor of Public Health Program, School of Health and Allied Sciences, Pokhara University, Pokhara, Nepal; 2 School of Health and Allied Sciences, Pokhara University, Pokhara, Nepal; 3 BP Koirala Institute of Health Sciences, Dharan, Nepal; University of South Florida, UNITED STATES

## Abstract

**Introduction:**

Nicotine dependence is an addiction to tobacco products caused by the chemical nicotine present in tobacco. 80% of premature deaths due to nicotine dependence come from low-and middle-income countries. Since most of the public health studies have focused solely on psychological and behavioral factors associated with tobacco smoking, this study aims to assess the nicotine dependence and stages of change of quitting smoking.

**Methodology:**

A community based quantitative cross-sectional study was conducted among 280 smokers aged 15–69 years in Bharatpur metropolitan, Nepal. A semi-structured and validated questionnaire was used during the face-to-face interviews. Nicotine dependence among participants was assessed using the six-item Fagerstrom Test for Nicotine Dependence (FTND). Chi-square test and multivariate logistic regression analysis were performed to assess the associations between variables at the significance level α = 0.05.

**Result:**

In the study population, the mean score of FTND was 5.15 ± 2.34. 33.9% participants had a high level of nicotine dependence and nearly half of the participants felt difficulty to refrain smoking even in No-smoking areas. Almost three out of ten respondents were prepared for smoking cessation. It was found that age group 20–39 years were more likely to have nicotine dependence (AOR 3.308, 95% CI = 1.039–10.534), those who initiated smoking before age of 15 were associated with a greater risk of nicotine dependence (AOR 3.68, 95% CI = 1.826–7.446), participants spending more on tobacco products (more than Rs 2400 monthly) were associated with an increased risk of nicotine dependence (AOR 4.47, 95% CI = 2.225–8.991), those who initiated smoking due to mental stress were more likely to have nicotine addiction (AOR 2.522, 95% CI = 1.004–6.028), and those who had no thought of quitting smoking were more associated with nicotine dependence (AOR 4.935, 95% CI = 1.458–16.699).

**Conclusion:**

Our study showed that high level of nicotine dependence is a major public health problem in low-and middle-income countries like Nepal. It also highlights that effective smoking cessation programs should be developed considering the level of nicotine dependence with more focus on early interventions of its associated factors.

## Introduction

Cigarettes smoking is one of the common risk factors for NCD [1]. It is practised by one-third of world’s population aged 15 years or older, among which 73% are in the developing countries [2]. Nicotine is a highly addictive chemical found in tobacco which makes it too difficult to stop smoking [3]. It produces physical and mood-altering effects on the brain that are pleasing to the person causing dependency [4, 5]. Nicotine addiction causes one person to die prematurely every six seconds, and 80% of them come from low-and middle-income countries [6]. In Nepal, according to a national representative survey, daily smoking prevalence is 48.4% and 28.7% for males and females (15 years and older) respectively [7]. Around 41 Nepalese die daily from tobacco-related diseases, which accounts to 27,000 deaths per year [8]. Yet, very few studies have been conducted on the assessment of nicotine dependence among smokers despite high smoking prevalence. Many public health studies have focused only on psychological and behavioral factors associated with tobacco smoking and nicotine dependence, a major barrier to smoking cessation, has always been a neglected issues so far [9].

The concept of ‘stage of change’ is at the core construct of the Transtheoretical model of behavior change (TTM) [10]. The TTM measures intention and behavior using stages of change: not planning to quit (pre-contemplation), intending to quit in the next six months (contemplation), and intending to quit in the next 30 days (preparation) [11]. This model is widely applied and often used to study determinants of smoking cessation. Evidence of validity of the TTM is strong with respect to smoking [12]. Having higher levels of nicotine dependence was significantly associated with a lower probability of smoking cessation a year later [13]. Precontemplation stage had did not contribute to predicting a transition from pre-contemplation to contemplation however it contributes significantly to predicting a transition from preparation towards action, which made it more difficult for them to adopt smoking cessation [14]. For effective implementation of smoking cessation programs, there is an urgent need to reduce dependence, identifying smokers attitude towards smoking cessation, and help smokers to progress through the stages of change. The aim of the study was to assess the nicotine dependence and stages of change of quitting smoking.

## Methodology

### Study design and population

A cross-sectional, quantitative study was carried out among 280 smokers aged 15 to 69 years in Bharatpur Metropolitan, Bagmati Province, Nepal from August 2019- March 2020. A sample size of 280 was determined based on 24% prevalence of life time nicotine dependence from a previous study conducted in China, at 95% confidence interval with estimated household of Bharatpur Metropolitan. Research subject eligibility assessment form was used to identify eligible participants for the study. Smokers aged 15 to 69 years who had smoked at least hundred cigarettes in their life-time and current smokers were eligible for the study but temporary residents, participants refusing to participate, and those who could not answer FTND questionnaire were excluded from the study. Simple random sampling technique was followed in which 9 out of 29 wards were selected using the Randomer software. The selected wards were 2, 7, 10, 11, 14, 16, 20, 21 and 26. Participants from each selected wards were determined proportionately based on household population. The calculated number of sample size from each ward was 36, 24, 58, 47, 19, 17, 14, 47 and 18 respectively. If a household does not have the any members meeting our research eligibility criteria, an adjacent household was chosen until a sample size of 280 was reached.

### Data collection and quality control

A face to face interview was carried out using interview schedule to gather the information. To ensure validity of the study, tool was developed using standard questionnaire on nicotine dependence from FTND, a standard tool developed by CDC/WHO. The FTND has been shown to have adequate validity and reliability and has been widely used in various settings [15–18]. The coefficient of constructed reliability of FTND was 0.73 in a study conducted in Singapore [15], and evidence of validity of the TTM was strong with respect to smoking [12]. An assessment of validity of our questionnaire was done by Item Objective Congruence (IOC), carried out by three public health specialists and a medical expert. In addition, translation and back-translation methods was used to further validate the questionnaire. To assess feasibility, order of questions, and comprehension pretesting was conducted among 28 participants. The Cronbach’s α was found at 0.72.

### Variables

The interview schedule included questions about socio-demographic characteristics such as age, sex, caste and education status. The participants also provided information about smoking behavior; duration of the smoking, age of initiation of smoking, use of smokeless tobacco products, whether they have ever tried to quit smoking, monthly expenditure on purchasing cigarettes, family history of smoking, influencing factors for initiating smoking, most often smoke place, health problems facing due to smoking, thought of quitting smoking and stage of smokers.

Dependent variables on nicotine dependence contained six standard questions of the Fagerstrom test for nicotine dependence (FTND) which determine the nicotine dependence of respondents and the total score ranged from 0–10. The scores were categorized as: 0–2 indicated a very low level of nicotine dependence, 3–4 indicated a low level of nicotine dependence, 5 indicated a medium level of nicotine dependence, 6–7 indicated a high level of nicotine dependence and 8–10 indicated a very high level of nicotine dependence.

### Data analysis

The data were first checked manually for completeness and then coded and entered into Epi-data (3.2 version) and was analyzed using IBM SPSS Statistics (20.0 version). Descriptive statistics was used to describe the study population which included mean, range, frequencies, and percentages. The first step in analysis was to find out the association between nicotine dependence and independent variables using chi-square test. Variables significant in Chi-square test with a p-value <0.05 were further analyzed by multivariate logistic regression.

### Ethical consideration

Ethical clearance was obtained from the Institutional Review Committee, Pokhara University. A formal letter of study approval (letter of cooperation) was obtained from Bharatpur Metropolitan City Office of Municipal Executive. Participants were fully informed about the purpose, nature and benefits of the research and full-informed consent was taken prior to the interview. Both written and verbal consents were also obtained. All data obtained from the participants were kept strictly confidential and used only for study purposes. We took an assent from minors and a consent from their guardians regarding our research. Since, smoking habit is quite sensitive issues and prevalent in Nepalese context, during interview, minors were asked without involving their guardian. Participants were well-informed about the tentative score of nicotine dependence and suggested to visit health facilities if required. In addition, each participant was counselled and motivated regarding the quitting of smoking cessation.

## Results

Out of 280 participants, more than two-third of participants (78.2%) were male and rest (21.8%) were female. The majority of participants (46.8%) were of age group 20–39 years followed by (30.0%) participants of age groups 40–59. The mean age of participants was 37.99 years (SD± 16.16). Majority of them (27.1%) were from relatively advantaged caste (Gurung, Newar) followed by (26.8%) upper caste (Brahmin, Chhetri). Most of them (55.0%) were Hindu. More than half of the participants (57.1) were married. Majority (46.6%) of participants had received secondary level education, majority (23.6%) of participants were labourer and 17.1% were involved in agriculture. Average monthly income of participants was RS 31441.96 and most of them (56.5%) had income less than NRS 25000. Socio-demographic characteristics of participants can be seen in Table 1.

**Table 1:** Socio-demographic characteristics (n = 280).

Variables	Number(n = 280)	Percent (%)
**Age**		
< 20 years	27	9.6
20–39 years	131	46.8
40–59 years	84	30.0
60+ years	38	13.6
**Sex**		
Male	219	78.2
Female	61	21.8
**Religion**		
Hindu	154	55.0
Buddhist	106	37.9
Christian	11	3.9
Muslim	9	3.2
**Ethnicity** ^**a**^		
Dalit	45	16.1
Disadvantaged janajati	59	21.1
Disadvantaged non-Dalit terai caste	17	6.1
Religious minorities	8	2.9
Relatively advantaged janajati	76	27.1
Upper caste	75	26.8
**Marital status**		
Married	160	57.1
Unmarried	105	37.5
Widowed	15	5.4
**Types of family**		
Joint	161	57.5
Nuclear	119	42.5
**Educational status**		
Illiterate	33	11.8
Literate (non-formal)	35	12.5
Basic level (<8 class)	50	17.9
Secondary Level (9–12 class)	130	46.4
Higher education	26	9.3
Postgraduate level	6	2.1
**Occupation**		
Unemployment	33	11.8
Business	27	9.6
Agriculture	48	17.1
Government employee	11	3.9
Non-government employee	44	15.7
Labour	66	23.6
Retired army	11	3.9
Student	33	11.8
Foreign employment	7	2.5
**Monthly income (n = 209)**		
<25000	118	56.5
25000–49999	58	27.8
50000–74999	23	11.0
>75000	10	4.8

^a^ Dalit (Damai, Kami, Sharki): Disadvantaged Janajati (Magar, Tamang, Rai/Limbu); Disadvantaged non-Dalit terai caste (Yadav and Thakur); Religious minorities (Muslim, chureto); Relatively advantaged Janajati (Newar, Gurung); Upper caste (Brahmin, Chhetri)

### Nicotine dependence

More than four out of ten (42.5%) participants used to start smoking within five minutes after waking up. Almost half of the participants (48.9%) felt difficult to refrain from smoking at places where it is forbidden. Nearly nine out of ten of participants (88.2%) reported that they hated morning time cigarette most to give up. More than four out of ten participants consumed eleven to twenty cigarettes in a day. More than six out of ten participants smoked more frequently during the first hours after waking and almost half (48.6%) participants smoked even when they were so ill and confined to bed most of the day. Based on FTND almost one-third participants (33.9%) had a high level of nicotine dependence Table 2.

**Table 2:** Nicotine dependence.

Variables	Number (n = 280)	Percent (%)
**Timing of Smoking after wake up**		
Within 5 minutes	119	42.5
After 5-30minutes	74	26.4
After 31–60 minutes	20	7.1
After 60 minutes and above	67	23.9
**Difficult to refrain from smoking in places where it is forbidden**		
Yes	137	48.9
No	143	51.1
**Cigarette you hate most to give up**		
The first in Morning	247	88.2
Any other	33	11.8
**Number of cigarette smoke/day**		
10 or less	107	38.2
11–20	121	43.2
21–30	52	18.6
**Smoke more frequently during the first hours after waking**		
Yes	172	61.4
No	108	38.6
**Smoke when you are so ill that you are in bed most of the day**		
Yes	136	48.6
No	144	51.4
**Level of Nicotine Dependence (ND)**		
Very low (0–2)	49	17.5
Low (2–4)	47	16.8
Medium (5)	44	15.7
High (6–7)	95	33.9
Very high (8–10)	45	16.1
Mean FTND	5.15 ± 2.34.	

Nicotine dependence was significantly associated with age (p = 0.001), marital status (p<0.001) and educational status (p = 0.001) Table 3.

**Table 3:** Socio-demographic characteristic and nicotine dependence.

Variable	Level of Nicotine dependence				Total	p-value
	Low		High			
	n	%	n	%		
**Age (years)**						
<20	17	63.0	10	37.0	27	0.001
20–39	50	38.2	81	61.8	131	
40–59	20	23.8	64	76.2	84	
60+	9	23.7	29	76.3	38	
**Sex**						
Male	77	35.2	142	64.8	219	0.559
Female	19	31.1	42	68.9	61	
**Marital status**						
Unmarried	51	48.6	54	54.1	105	**<**0.001
Married	45	25.7	130	74.3	175	
**Type of family**						
Nuclear	46	38.7	73	61.3	119	0.185
Joint	50	31.1	111	68.9	161	
**Ethnicity**						
Upper caste	20	26.7	55	73.3	75	0.104
Lower caste	76	37.1	129	62.9	205	
**Religion**						
Hindu	52	33.8	102	66.2	154	0.840
Non-Hindu	44	34.9	82	65.1	126	
**Monthly income (in Nepalese rupees)**						
<20000	34	36.2	60	63.8	94	0.458
≥20000	36	31.3	79	68.7	115	
**Educational status**						
Below secondary	28	23.7	90	76.3	118	0.001
Secondary or above	68	42.0	94	58.0	162	
**Employment status**						
Unemployed	27	25.0	81	75.0	108	0.280
Employed	69	40.1	103	59.9	172	

The overall median age at smoking initiation was 16 and the median duration of cigarette smoking was 15 years. Almost 35% smokers had a family history of smoking. Three out of ten (28.6%) respondents consume both smoke and smokeless tobacco products. Age at smoking initiation (p<0.001), duration of smoking (p<0.001) and most often smoke place (p<0.001) were found to be highly significant with the level of nicotine dependence. Monthly expenditure on smoking (p = 0.001), family story of smoking (p = 0.011), influencing factors for initiating smoking (p = 0.010), health problem due to smoking (p = 0.010) and stage of smokers (p = 0.004) were also found statistically significant with nicotine dependence level of respondents. Smoking behavior related characteristics and nicotine dependence characteristics of the participants can be seen in Table 4.

**Table 4:** Smoking behavior related characteristics and nicotine dependence.

Variable	Level of Nicotine dependence				Total	p-value
	Low		High			
	n	%	n	%		
**Age at smoking initiation**						
<15 years	30	22.6	103	77.4	133	**<**0.001
≥15 years	66	44.9	81	55.1	147	
**Duration of smoking**						
<15 years	25	67.6	12	32.4	37	<0.001
≥15 years	71	29.2	172	70.8	243	
**Monthly expenditure on smoking (in Nepalese rupees)**						
<2400	62	44.0	79	56.0	141	0.001
≥2400	34	24.5	105	75.5	139	
**Family history of smoking**						
Yes	24	24.5	74	75.5	98	0.011
No	72	39.6	110	60.4	182	
**Consuming smokeless tobacco**						
Yes	22	27.5	58	72.5	80	0.130
No	74	37.0	126	63.0	200	
**Restarting of smoking**						
Yes	24	24.5	74	75.5	76	0.582
No	72	39.6	110	66.7	204	
**Most often smoke places**						
Home	25	20.5	97	79.5	122	<0.001
Friend place	45	48.4	48	51.5	93	
Other places	26	40.0	39	60.0	65	
**Influencing factors for initiating smoking**						
Peer pressure	73	38.6	116	61.4	189	0.010
For experiment	8	53.5	7	46.7	15	
Imitation	3	15.0	17	85.0	20	
Mental stress	12	21.4	44	78.6	56	
**Health problem due to smoking**						
Yes	9	18.4	40	81.6	49	0.010
No	87	37.7	144	62.3	231	
**Ever thought of quitting smoking**						
Yes	24	25.0	72	75.0	96	<0.001
No	16	8.7	168	91.3	184	
**Quitting attempts of smoking in past 24 hours**						
Yes	24	20.0	96	80.0	120	0.018
No	16	10.0	144	90.0	160	
**Stage of smokers**						
Pre-contemplation	59	28.6	147	71.4	206	0.004
Contemplation	17	48.6	18	51.4	35	
Preparation	20	51.3	19	48.7	39	

In multi-variate logistic regression analysis, after adjusting for potential confounding factors, five variables were found to be significantly associated with nicotine dependence: age of the participants (years), age at smoking initiation (years), monthly expenditure on smoking, influencing factors for initiating smoking and ever thought of quitting smoking. Participants of age group (20–39) years were 3.30 (95% CI = 1.039–10.534) times more likely to have nicotine dependence in comparison to the participants less than 20 years. Those who initiated smoking before the age of 15 were 3.68(95% CI = 1.826–7.446) times more likely to nicotine dependence than who initiated after age of 15. Those who spent more than Rs 2400 per month in smoking had 4.47(95% CI = 2.225–8.991) times higher chance of nicotine dependence than those who spent less. Those who initiated smoking due to mental stress was 2.52(95% CI = 1.004–6.028) times more likely to develop nicotine dependence than those who started on peer pressure. In addition, participants who had no thought of quitting smoking were 4.94(95% CI = 1.458–16.699) times more likely to have nicotine dependence than respondent who had thought of quitting smoking. The results of bivariate and multivariate logistic regression analysis between nicotine dependence and selected variables can be seen in Table 5.

**Table 5:** Bivariate and multivariate logistic regression analysis between nicotine dependence and selected variables.

Variable	UOR(95%CI)	AOR(95%CI)
**Age(Years)**		
<20	1	1
20–39	2.754(1.169–6.488)	**3.308(1.039–10.534)**
40–59	5.440(2.149–13.768)	1.922(0.425–8.688)
60+	5.478(1.858–16.153)	0.766(0.129–4.527)
**Marital status**		
Unmarried	1	1
Married	2.728(1.636–4.549)	1.859(0.754–4.581)
**Educational status**		
Below secondary	2.325(1.373–3.937)	1.621(0.681–3.854)
Secondary or above	1	1
**Age at smoking initiation**		
≥15 years	1	1
<15 years	2.798(1.662–4.708)	**4.134(1.988–8.598)**
**Duration of smoking**		
<15 years	1	1
≥15 years	5.047(2.404–10.597)	0.997(0.346–2.879)
**Monthly expenditure on smoking (in Nepalese rupees)**		
<2400	1	1
≥2400	2.424(1.455–4.036)	**4.270(2.091–8.717)**
**Family history of smoking**		
No	1	1
Yes	2.018(1.167–3.491)	1.449(0.745–2.821)
**Most often smoke places**		
Other places	1	1
Home	2.587(1.333–5.019)	2.312(0.972–5.495)
Friend Places	0.711(0.374–1.351)	0.813(0.356–1.856)
**Influencing factors for initiating smoking**		
Peer pressure	1	
For experiment	0.551(0.192–1.583)	0.331(0.082–1.344)
Imitation	3.566(1.010–12.595)	1.916(0.422–8.692)
Mental stress	2.307(1.143–4.657)	**2.522(1.000–6.360)**
**Health problem due to smoking**		
No	1	1
Yes	2.685(1.243–5.802)	2.094(0.814–5.384)
** Ever thought of quitting smoking**		
Yes	1	1
No	3.500(1.755–6.979)	**4.935(1.458–16.699)**
** Quitting attempts of smoking in past 24 hours**		
Yes	1	1
No	2.250(1.136–4.456)	1.325(0.540–3.253)
**Stage of smokers**		
Preparation	1	1
Contemplation	1.115(0.447–2.777)	0.609 (0.161–2.307)
Pre-contemplation	2.623(1.307–5.264)	1.276(0.410–3.975)

## Discussion

This study was carried out to find out the level of nicotine dependence and quitting stage of smokers. Nepal is a country where smoking prevalence is high and smoking advertising and restrictions are very limited. Nicotine dependence has multiple public health implications. First, nicotine dependence is a major force driving the urge to smoke, making it very difficult to quit. Second, nicotine dependence has a high degree of co-morbidity with certain psychiatric conditions such as substance abuse and mood disorders. Third, smoking is the most common risk factor for ill health, which directly affects the respiratory and cardiovascular systems and causes premature death [19, 20]. Due to these adverse effects associated with nicotine dependence, it is necessary to assess the level of dependency and stage of change to better understand mechanism. Furthermore, it would also help in successful implementation of smoking cessations programs.

Our study revealed that the level of nicotine dependence was high in 33.9% and very high in 16.1% smokers which is similar to the study conducted in Vietnam [19], South Africa [21]. The study conducted in three districts of Nepal revealed that FTND was very low in 31.9%, low in 17.1%, medium in 30.3% and high in 20.4% [3]. This reflects the level of nicotine dependence was higher in our study as compared to their study. This inconsistency might be because of the different pattern of tobacco smoking. It might also depend on the different cut-off points in FTND.

In our study, mean FTND score was found to be 5.15 ± 2.34. This score is more or less similar to the study of Saha et al. (6.47 ± 2.38) [5], Kõks et al. (4.1 ± 2.4) [19]. The level of dependence was also calculated to understand the percentages of smokers who have a high risk to health due to dependency. This suggests that smokers having high dependency are more vulnerable to various NCD’s in a future stage of life. Our study showed that a particular age group 20–39 years was associated with a higher risk of nicotine dependence which highlights the significance of stopping smoking as early as possible. There was no significant association between sex and nicotine dependence in this study which is similar to the study conducted in three districts of Nepal. Contrary to this study, there was an association between sex and nicotine dependence a study conducted in India [22], China [9], and Spain [23]. This may be due to the socio-cultural differences; female smokers are not generally well accepted in Nepalese context. Age of smoking initiation was an important predictor of nicotine dependence in our sample revealing that respondents, who started smoking before the age of 15 were more likely to have nicotine dependence than those who initiated after the age of 15. This result is consistent with the results of a study conducted in India that reported that the early onset of smoking is associated with a higher risk of nicotine dependence [24]. Thus, health promotion and education campaigns addressing the adverse health consequences of smoking should specially target individuals younger than 15 years old. In addition, laws and regulations prohibiting underage sales of cigarettes should be formed and implemented. Furthermore, this study showed that smokers with high monthly expenditure on smoking had higher level of nicotine dependency. Similarly, among the several factors which influenced to initiate smoking, people who reported mental stress to be one, later had high degree of nicotine dependence. Thus, it is very important to address mental stress on time to decrease initiation of smoking and nicotine dependency. Our study showed that almost 35% smokers had a family history of smoking. It had been shown that smoking habit run in families [24]. When parents are the ones who smoke, it is very hard for them to persuade their children to avoid or stop doing something that they see their parents do.

This study revealed that the 73.6% of the respondents were in pre-contemplation stage, 12.5% were in the contemplation stage and 13.9% were in the preparation stage. However, no significant association was illustrated between stage of smokers and level of nicotine dependence. As majority of the respondents were not sure about they could quit which demands a stronger counselling service. Furthermore, special tobacco cessation program and strategies are required for individuals who report nicotine dependence and are in preparation and contemplation stage of tobacco cessation. The baseline information related to nicotine dependence and stage of change of cigarette smokers are very crucial for policy makers and the government to implement effective tobacco control programs in Nepal.

## Conclusion

Our study demonstrates that nicotine dependence was highly substantial with low level of attempts to quit smoking among Nepalese population. Next, smoking cessation programs should be designed and implemented taking into account the level of nicotine dependence and the stage of change of the smokers. The high nicotine dependence identified throughout this study highlights a major public health problem. There is a need for further research and study also indicates that there is a high demand for supportive counselling for quitting smoking.

## Supporting information

S1 File Complete dataset 1. (RAR)
